# Enhancing and expanding intersectional research for climate change adaptation in agrarian settings

**DOI:** 10.1007/s13280-016-0827-0

**Published:** 2016-11-22

**Authors:** Mary Thompson-Hall, Edward R. Carr, Unai Pascual

**Affiliations:** 1 International START Secretariat, 2000 Florida Avenue N.W., Suite 200, Washington, DC 20009 USA; 2 IDCE, Clark University, 950 Main Street, Worcester, MA 01610-1477 USA; 3 Basque Centre for Climate Change (BC3), Edificio Sede Nº 1, Planta 1ª, Parque Científico de UPV/EHU, Barrio Sarriena, 48940 Leioa, Spain; 4 IKERBASQUE, Basque Foundation for Science, Maria Diaz de Haro 3, 6 floor, 48013 Bilbao, Spain; 5 Department of Land Economy, University of Cambridge, 19 Silver St., Cambridge, CB3 9EP UK

**Keywords:** Agriculture, Climate change adaptation, Gender, Identity, Intersectional, Vulnerability

## Abstract

Most current approaches focused on vulnerability, resilience, and adaptation to climate change frame gender and its influence in a manner out-of-step with contemporary academic and international development research. The tendency to rely on analyses of the sex-disaggregated gender categories of ‘men’ and ‘women’ as sole or principal divisions explaining the abilities of different people within a group to adapt to climate change, illustrates this problem. This framing of gender persists in spite of established bodies of knowledge that show how roles and responsibilities that influence a person´s ability to deal with climate-induced and other stressors emerge at the intersection of diverse identity categories, including but not limited to gender, age, seniority, ethnicity, marital status, and livelihoods. Here, we provide a review of relevant literature on this topic and argue that approaching vulnerability to climate change through intersectional understandings of identity can help improve adaptation programming, project design, implementation, and outcomes.

## Introduction

Resilience frameworks examine the capability of a system to flexibly maintain the ability to respond to anticipated and unanticipated changes and stressors without compromising its ability to function or its ability to react and transform (Walker et al. 2006; Adger et al. 2011). In the context of climate change, the collective ability of social actors and ecological components to adapt to particular impacts shapes the overall resilience of social–ecological systems where adaptation is understood as the “process of adjustment to actual or expected climate and its effects” (IPCC IPCC 2014, p. 5). In order to better understand this collective ability and to identify opportunities for improving adaptation strategies and interventions, it is necessary to look more closely at the composition of vulnerability for particular places, people, and ecosystems. This composition has been identified as reflecting exposure to change, sensitivity (i.e., susceptibility to be harmed by change), and adaptive capacity (i.e., possessing the tools and resources for dealing with change as well as the ability to use those for adaptation) (Nelson et al. 2007; IPCC 2014). Investigation of the complex origins of differential types and degrees of exposure, sensitivity, and adaptive capacity associated with diverse groups of people and unique locations (including gendered dimensions) has a deep-rooted history that spans a number of disciplines. Take, for example, the early 1990s work of Liverman (1990) that explored different dimensions of vulnerability to global environmental change. Also, notably, the political ecology explorations of Peet and Watts (2004), where critical examination was given to a compilation of cases that illuminate structural sources of differential environmental vulnerabilities. Early examples from hazards studies can be found in the work of Adger (1999) on social vulnerability to climate change and extremes, or the work of Turner et al. (2003) on differential vulnerabilities to hazards. Feminist geographers such as Joni Seager (1993), Fordham (1998), and host of others have further pushed a critical gendered lens within this scholarship.

Today, for agrarian settings in the Global South, predictions regarding the impacts of climate variability and change are particularly alarming. Uncertain futures of increased variability in the amount, duration, and distribution of precipitation will likely produce place-specific challenges, such as lengthier droughts, more intense flooding, and increasingly widespread pests and diseases (chapters 22 and 24 in IPCC 2014). At times, these impacts will play out as *distinct* vulnerabilities, where portions of the population have a different exposure to a particular climate stressor (Carr and Thompson 2014). In other cases, these impacts may produce *differentiated* vulnerabilities, where an entire population shares the same exposure to a climate stressor, but have different sensitivities and adaptive capacities with regard to that stressor (Carr and Thompson 2014). It is the deeper unpacking of how these differences in sensitivities and adaptive capacities come about, how they are sustained, and what the implications of these are for resilience in overall dynamic systems that are a critical point of exploration for advancing the body of knowledge on climate change adaptation.

While current literature on supporting and facilitating adaptation in agrarian settings increasingly acknowledges that complex social factors can influence how impacts of climate change affect different people in particular ways (Adger 2010; Agrawal 2010; Ribot 2011), within practice the inclusion of these factors into research and initiatives focused on adaptation remains superficial and based on outdated assumptions about identity and its associations with vulnerability (Carr and Thompson 2014). For example, those that presume that only men are the farmers in families or communities (Demetriades and Esplen 2010) or that blindly promote the idea that in any given agrarian context women tend to be the most poor and most vulnerable (Jackson 1998; Chant 2010). Intersectional framings, however, give deeper attention to multiple facets of farmer identities and the way these facets come together to influence vulnerability of different people. Such framings can open new opportunities for building more robust understandings of dynamic assemblages of power and institutions and how these assemblages shape sensitivity and adaptive capacity. We seek to untangle how intersectional framings relate to, and could be more effectively used to support adaptation planning and programming. A goal of this paper therefore, is to provide a review of this topic for researchers and practitioners working outside of the social sciences, or outside of the fields of feminist and gender studies, to better gauge how perspectives from these lines of work can be utilized to support more legitimate and useful adaptation actions and, in turn, increase resilience of agroecosystems in the Global South.

## Gender and adaption programming

Exposure is a function of one’s location and environment, which in turn is shaped by historically developed social institutions, both formal and informal. Sensitivity and adaptive capacity are largely dominated by social markers that influence dimensions like access to and control over resources, type, and location of livelihood activities, as well as meanings and values assigned to different resources and activities. Literature on vulnerability and adaptation in agrarian settings in the Global South has tended to focus on one such marker, gender, as a primary category of difference that influences a person’s vulnerability and their ability to adapt (see Carr and Thompson 2014 for a recent summary of such work). This work generally revolves around three core themes: (i) lack of women’s inclusion in decision making (Mehra and Hill Rojas 2008; Dankelman and Jansen 2010), (ii) gendered inequalities in access to land and land tenure (Brody et al. 2008; Quisumbing and Pandolfelli 2008; Djoudi and Brockhaus 2011; FAO 2011), and (iii) gendered agricultural practices and crop choices (Barry and Schlegel 1982; Arndt and Tarp 2000; Carr 2008; Ravera et al. 2016). Such themes have emerged out of a long history of influence from the rich collection of knowledge generated within interdisciplinary feminist studies on development policy, practice, and research since the 1970s.

Feminist studies have grown from activism and theorizing against discrimination and subjugation of women, to exploring the nature and implications of socially constructed roles of women and men in society. Increasingly, since the 1970s and 1980s, this scholarship has increasingly engaged with post-structural methodologies, applying lessons learned from gender relations to wider understandings of identity through the recognition of diverse intersections of power and identity *within and across* different groups of women and men themselves (Valentine 2007).

Throughout this intellectual evolution, gender issues have shifted from the margins to the core of development agendas. Today, the UN calls for “gender mainstreaming” and the fifth Sustainable Development Goal calls for achieving gender equality (UN DESA 2015). But, in practice, this work does not often progress beyond the gathering of sex-disaggregated data toward critical interrogation of the more complex impacts of intersecting dimensions of identity (including gender) on a person or group’s vulnerability. The reasons for this are multiple and often structural. To investigate this, it can be useful to take a step back and examine some of the history of how we got to this point.

In the late 1980s, at the same time that feminist studies and a focus on gender were becoming integrated into international development agendas, the introduction of the influential set of ideas, collectively labeled “sustainable development,” brought the two unique but related projects of international development and environmental protection together. Most recently, sustainable development has come center stage once again with the establishment of the Sustainable Development Goals (SDGs), part of the 2030 Agenda for Sustainable Development adopted by the UN in 2015 (UN 2016).

Feminist studies have, in parallel, engaged with concepts of sustainability and environmentalism in various ways, perhaps most influentially with the emergence of ecofeminism in the 1970s and into the 1990s, especially following the “Women and Life on Earth: A Conference on Eco-Feminism in the Eighties” (Caldecott and Leland 1983) following the meltdown at the Three Mile Island nuclear plant in the USA. Ecofeminism links masculinist conceptualizations of nature and women with subjugation, and has tended to attribute unique and inherent connections between women and the natural environment, connections not possessed by men (Mies and Shiva 1993). Others, however, have endeavored instead to highlight the substantial material basis for this women-environment link such as Bina Agarwal did (1992), with her investigation of the situation of women and development in India with regard to power, property, and knowledge.

Current works on the connections between gender, agriculture, environment, and development have largely moved past ecofeminism’s original essentializing connections between women and nature (Cornwall et al. 2007; Leach 2007). This work has built on that of Agrawal and others and has moved toward investigating how the roles of women in agrarian settings often position them in unique relationships with agroecosystems in terms of resource use, land ownership, and tenure (e.g., Babugura et al. 2010; Demetriades and Esplen 2010; Sultana 2010, 2014; Tatlonghari and Paris 2013). However, to date, the bulk of this work continues to rely heavily on inquiry framed around simple conceptualizations of gender. These conceptualizations most often take one of the two forms, those that conflate “gender” to mean “women,” i.e., the “add women and stir” conceptualization (Harding 1995), or those that conflate “gender” to mean “men versus women.” Each of these conceptualizations problematically holds the potential to reduce an incredibly diverse dimension of identity into a uniform box-ticking opportunity. As Cornwall and Rivas (2015, p. 399) put it: “Relegating gender to a descriptive home is an attractive option for those who want to talk the gender talk in the absence of real debates about power.” Power is often missing from these conceptualizations of gender, which mask questionable assumptions that can be observed through, for example, the continued use of the categories of “men” and “women” as explanatory categories for variations in vulnerability without supporting empirical evidence. In such cases, outdated, a priori assumptions of *men/less vulnerable, women/more vulnerable* are relied upon without further investigation of nuanced sources of vulnerability (Pelling and High 2005; Adger 2006; Paavola and Adger 2006; Reid and Vogel 2006). The principal problem for adaptation programming is that this framing only scratches the surface about the roles and responsibilities that yield observed patterns of vulnerability to climate change. Moreover, this reliance on superficial categories can lead to equally superficial results that fall short of informing more effective adaptation strategies.

A large portion of today’s climate change adaptation and development work attempts to capture a broad assemblage of social characteristics such as gender, age, income, education level, land ownership, and others related to formal and informal institutions, in their vulnerability analyses (Fisher 2014; Winowiecki et al. 2014). However, such data collection often still relies upon a priori assumptions about what different identities mean in a given place, and what vulnerabilities those identities produce in particular places. This tendency can be seen in studies that employ approaches and methods ranging from rapid rural appraisals (Patt et al. 2009; Winowiecki et al. 2014) using tools including community surveys, participatory focus groups, household level surveys, to randomized control trials (Banerjee and Duflo 2009; Karlan and Zinman 2011) that principally gather primary information from local populations and key actors. Such research serves important purposes, for example, by highlighting important instances of inequality and marginalization. However, when carried out without strategic attention to intersecting influences of identity, the explanatory value of such methods for understanding differential vulnerabilities can only fall short of what could potentially be accomplished through engagement with more recent feminist scholarship that fosters exploration of multiple intersections of identities, knowledge, power, and agency. Although there are a number of highly relevant and impactful facets of this scholarship, in the next section we take the opportunity to examine one, intersectionality, more closely with regard to its potential contributions to adaptation in agrarian settings.

## Intersectionality and its relevance for adaptation to climate change

An opportunity exists for strengthening a growing engagement of adaptation research with cross-disciplinary feminist scholarship on intersectionality. *Intersectionality* as a concept first originated in the late 1980s and early 1990s through discontent with what some feminist scholars perceived as a privileging of white middle class women’s perspectives in the feminist movement over those of women of color or poor women (Hooks 1984; Crenshaw 1991; Kaijser and Kronsell 2014). Davis (2008, p. 68) defines it as “the interaction between gender, race and other categories of difference in individual lives, social practices, institutional arrangements, and cultural ideologies and the outcomes of these interactions in terms of power.” Lykke (2011, p. 207) gives a less structured definition of the concept as “a nodal point… an open ended framework for comparing different feminist conceptualizations of intersecting power differentials, normativities, and identity formations.”

Uptake of intersectional conceptualizations within research on global climate change is only just beginning. Kaijser and Kronsell (2014) provide a broad theoretical overview of how intersectional understandings relate to climate change and introduce a number of sensitizing questions that can be implemented to critically approach power relations within climate change research. These questions, such as “Are there any observable explicit or implicit assumptions about social categories and about relations between social categories?” and “Are any other aspects of identity neglected or deemed insignificant?” seek to go beyond conventional framings to help illuminate relevant aspects of identity that may currently be overlooked (Kaijser and Kronsell 2014, pp. 429–430).

Intersectional framings recognize that it is the *roles* and *responsibilities* associated with particular identities which shape who does what, how they do it, when they do it, with what resources, and to what ends. However, the identities and associated roles and responsibilities shift depending on the activity at hand, and the identities that activity mobilizes. One example from Carr and Thompson (2013) comes from Mali where even a gender analysis that moved past outdated assumptions of women/vulnerable, men/less vulnerable was shown to fall short of the more comprehensive understandings of vulnerability that could be achieved with intersectional methods.^1^ In this case, a conventional (sex-disaggregated) gender analysis would highlight how a focus on rain-fed agriculture by men would make them more vulnerable to variable precipitation than would be women farmers whose primary focus is hand-irrigated gardens. Yet, if broader convergences of identity markers are taken into account such as the intersection of gender and seniority, a more nuanced picture of vulnerability is revealed. Such an analysis would show that junior men are more reliant on sales of surplus of rain-fed crops than are senior men, therefore making the junior men more vulnerable to fluctuations in precipitation (Carr and Thompson 2013). Further, while both junior and senior women participate in hand-irrigated gardening, senior women are more dependent than are junior women on added market sales of rain-fed peanuts to bolster their earnings from their home gardens. Junior women only use peanuts to supplement their household’s subsistence. This example illustrates that, in contrast to the situation with men, senior women may indeed be more vulnerable to variable precipitation than are junior women, an illustration that highlights the potential insights gained from pushing beyond conventional man/woman binaries.

Through utilizing an intersectional lens, such nuanced approaches can help to better target stress-specific roles and responsibilities, and therefore build tailored understandings of vulnerability that are specific to the stressor and one or more specific activities (e.g., farming of rain-fed crops), making it easier to identify appropriate adaptation-based policy interventions. Pursuing intersectional investigations into the vulnerabilities of a given population without explicit goals and objectives could yield large, unmanageable bodies of data. However, when directed at answering specific vulnerabilities to specific stressors, such as climate change-related impacts, these framings can help to more effectively identify situational aspects such as informal institutions, e.g., social norms that may be hindering climate change adaptation. Further, these framings can serve to help identify opportunities for changing those aspects to facilitate adaptation. Therefore, we contend that investing in gathering information at such enhanced detail is not an unnecessary burden, but instead it would help to design more streamlined and replicable adaptation strategies across regions.

## Discussion

While it is not difficult to envision the value that such understandings may hold for localized efforts relating to climate change adaptation, when viewed in isolation, such applications of intersectional approaches may seem to contribute little to broader efforts at enhancing resilience at larger social–ecological scales. On the other hand, when viewed as part of a vast network of integrated system components, we see such applications as representing a collection of steps toward greater capacity and increased flexibility. As argued by Nelson et al. (2007, p. 399) when addressing the relationship between adaptation and resilience frameworks:

“a resilience framework is concerned with context, feedbacks, and connectedness of system components. This is a fundamental difference with the adaptation to environmental change literature that is focused on actors. … reconciliation of actor-and system-oriented approaches represents a major challenge… actor-based analysis looks at the process of negotiation and decisions, and the systems-based analysis examines the implications of these processes on the rest of the system.”

This relationship between reduced vulnerability via increased adaptive capacity or reduced sensitivity in one part of a system and increased resilience of the system as a whole, however, is not necessarily a simple or linear one (Walker et al. 2006; Nelson et al. 2007). Therefore, the challenge is to increase our understanding about the way intersectionality can be used as a key contributor and supporter of the growing toolbox of strategies for reducing localized vulnerabilities to climate change, and for bolstering flexible strategies of transformative adaptation and resilience of agrarian systems across scales.

### Engaging intersectionality in agrarian settings

**Fig. 1:**
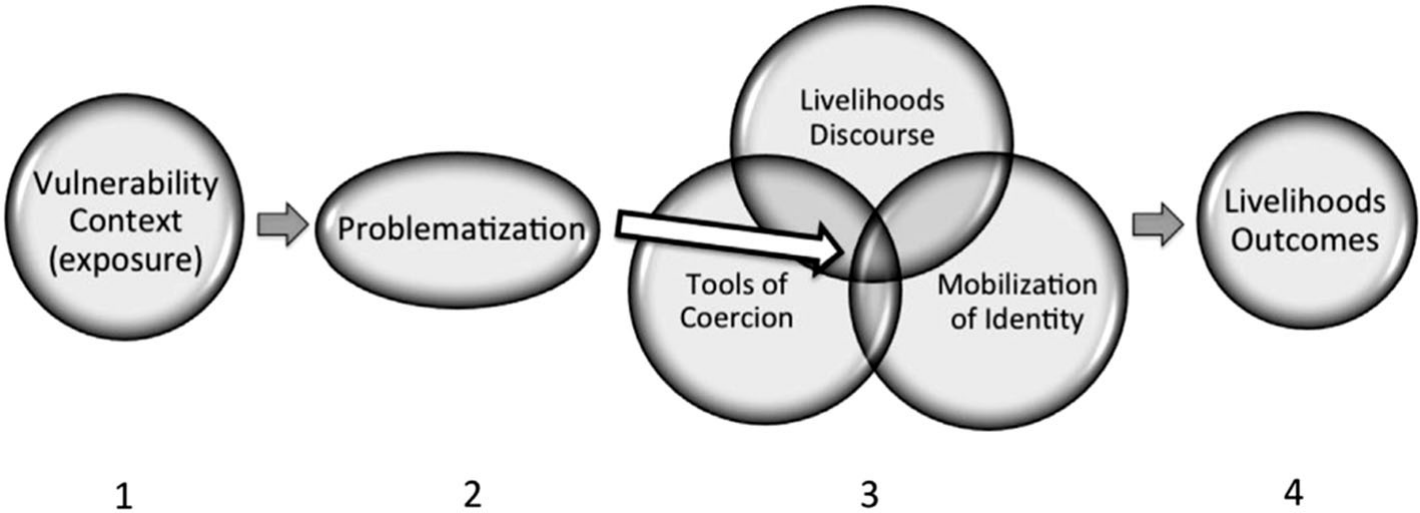
*Livelihoods as Intimate Government* (*LIG*) four-step process. (1) Establishing current vulnerability context. (2) Identifying instances where logic and legitimacy of livelihoods strategies are questioned by those who participate in them. (3) Opens insights into the nexus of livelihoods strategy formation. (4) Leading to explanatory interpretation of livelihoods outcomes (Carr 2014: 114)

Applied within localized agrarian settings, intersectional approaches offer ways of understanding how social dimensions of identity (encompassing gender) are bound up in systems of power and social institutions (both formal and informal) to shape situation-specific interactions between individual farmers, households, and agroecosystems. These intersections result in unique and dynamic adaptation needs. At this (or any other) scale, there is no one approach or defined set of methods that represent best practices for seeking intersectional understandings of vulnerability relating to climate change. We agree with Davis (2008), Lykke (2011), and Kaijser and Kronsell (2014) who stress that the complexity of intersectionality demands a multiplicity of disciplinary perspectives and should not be boxed in by any one set of priorities or uses. That being said, there are novel approaches and methods that support intersectional understandings and are applicable to issues that link climate change, livelihood strategies, and agroecosystem management. One such approach is based on the *Livelihoods as Intimate Government* (LIG) framework and methodological approach (Carr 2013, 2014). LIG encompasses non-material factors (for example, societal norms and other informal institutions that shape and are embodied through livelihoods decisions) that influence the exposure, sensitivity, and adaptive capacity of certain groups and individuals facing uncertain climate futures. In so doing, LIG offers an explicit method for gathering empirical data needed to support an intersectional framing. The method is composed of a four-step approach for capturing the various motivations for livelihoods decisions, from material provisioning to the maintenance of social status, without making a priori assumptions as to which motivation is of highest importance (Fig. 1; Carr 2014).

This approach was developed to interrogate livelihoods decisions in agrarian settings in Ghana. It has since been applied to livelihoods decisions in Mali (Carr et al. 2015a; Carr and Owusu-Daaku 2016), Senegal (Carr et al., 2016), and Zambia (Carr et al. 2015b) as part of efforts to better understand the needs for various forms of weather and climate information in agrarian settings. In all cases, LIG enabled nuanced understandings of the complex factors that intersect to shape the everyday decisions of different people living within the context of a changing climate. Multiple methods are encompassed within this approach including literature review, semi-structured interviews, household surveys, and participant observation.

Currently, research is emerging that integrates a deeper interrogation of power and intersectionality, with a focus on the convergences of social–ecological dimensions. For example, in a recent collection of political ecology works on water resource issues and environmental change (Buechler et al. 2015). Kambic (2015) uses discourse analysis to explore power and influence at the intersections of gender social class and geographic location in the context of urban Los Angeles water systems. Another example from human geography and political ecology can be found in the work of Nightingale (2011) who uses intersectional framings to explore symbolic ideas of difference and how these ideas translate into material realities through everyday decisions and behaviors that involve the use of agricultural and forest resources in Nepal. More recently, Evans (2016) used a feminist ethnographic methodology encompassing purposive and snowball sampling, semi-structured interviews, and participatory workshops to examine the intersections of gender, religion, ethnicity, marital position, status, and generation with socio-ecological change to better understand shifting inheritance practices among the Serer ethnic group in Senegal. Regarding climate change adaptation or resilience of systems specifically, calls for more meaningful engagement with intersectionality work in political ecology and feminist geography are increasing (Sultana 2014); however, the application of intersectional approaches situated in social-ecological perspectives remains quite thin.

#### Scaling-up intersectional understandings

Clearer understandings of localized realities within specific agrarian settings, e.g., of a given farming community in a given locality, can be used to better understand how a diversity of settings interacts with one another within a broader agrarian landscape. To the authors’ knowledge, this type of “scaling-up” has, to date, not been undertaken with specific regard to linking intersectional understandings with broader information on vulnerability and ecosystem processes across interlinked landscapes.

**Fig. 2:**
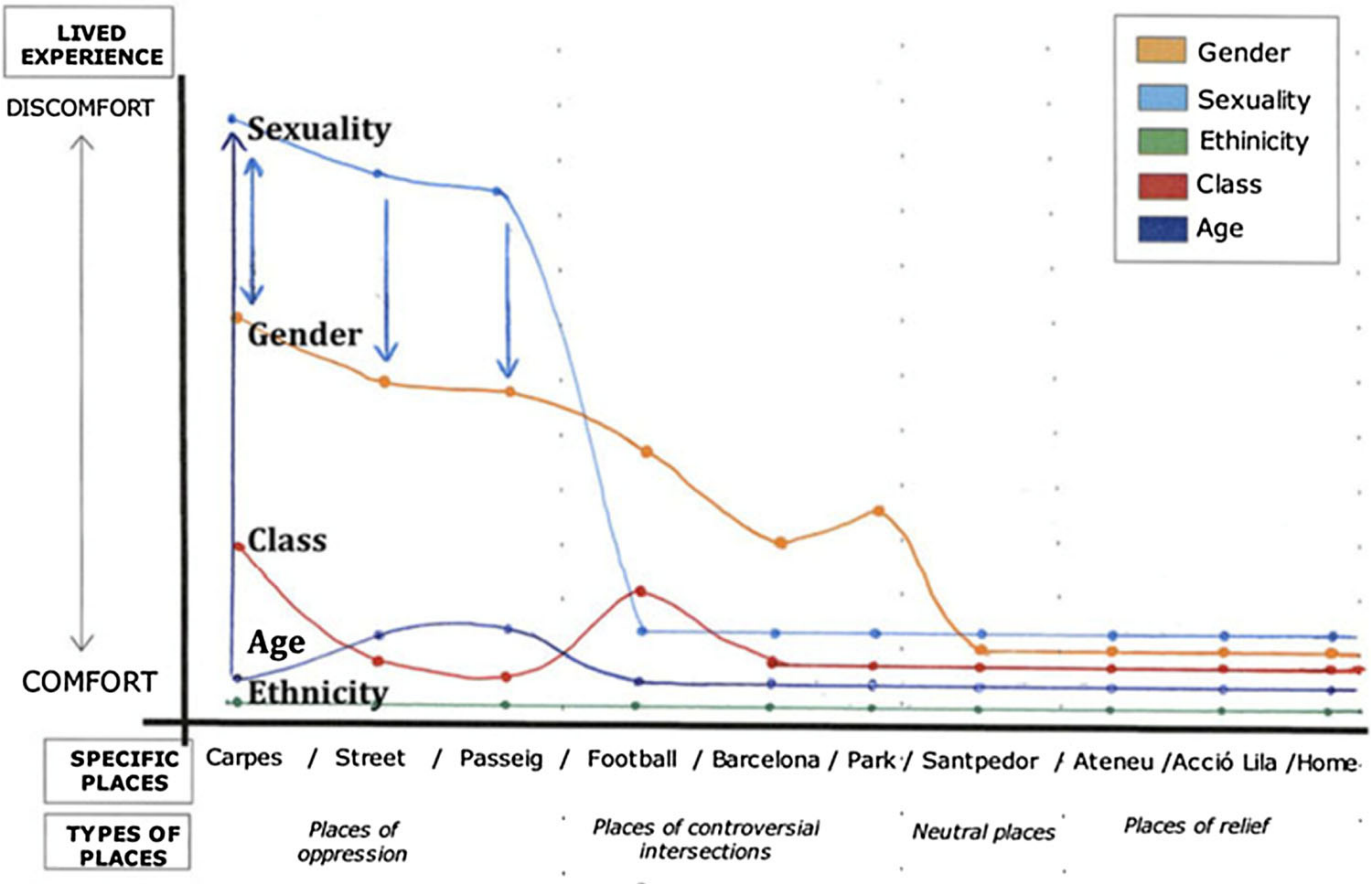
Relief Map for a girl living in Manresa, Catalonia, illustrating her lived experience of integrated dimensions of power, space, and oppression/privilege as she moves through different areas of her hometown (Rodó-de-Zárate 2014, p. 929)

Recent work that makes use of *participatory* geographic information systems (PGIS), *critical* GIS (Harvey et al. 2005), and *feminist* GIS (Elwood 2008) may offer potentially fruitful areas of exploration for this type of integration. These are a result of increased concern in the 1990s over unequal power relations and access, and privileging of certain masculine epistemologies within spatial information and geographic information systems that can overshadow more marginalized or less powerful perspectives (Aitken and Michel 1995). Examples of such work can be found within literature on planning and governance (McCall 2003) and for mapping the social values of local people with regards to natural resources (Tyrväinen et al. 2007; Bernard et al. 2011; Villamor et al. 2014). Kwan (2002a, b) advanced this work with respect to gender and feminist studies by conceptualizing how *critical* and *participatory* GIS could be merged with feminist epistemologies to better integrate quantitative and qualitative data within spatial visualizations, as well as for bringing more comprehensive perspectives to issues of political and social change. Since then, *feminist* GIS has emerged as a dynamic research line (Sui and DeLyser 2012). This line is inclusive of works such as those focused on impacts of GIS in women’s lives (McLafferty 2005) and gender and agriculture (Harman 2013). We see opportunities for advancing the scope of such integrated and spatially explicit methods that could encompass intersectional understandings within broader scale analyses of farmer vulnerability and of farmer understandings of agroecosystems. This type of work could build not only on so-called ‘relief maps’ that highlights both the traditional use of the word “relief” in mapping, but also the removal of anxiety or pain and which can be used to explore spatial mapping applications of intersectionality (See Fig. 2 for an example from Rodó-de-Zárate 2014). These maps have three dimensions, the social (power) dimension, the geographical dimension, and the psychological dimension and serve to illustrate how space, power, and privilege/oppression are connected.

Another research area of interest here is bridging intersectional gender approaches and the ecosystem services framework, especially as traditional GIS mapping of ecosystem services is now being complemented with mapping of beneficiaries and producers of such services. Further, emerging ecosystem services framings, such as that arising from the Intergovernmental Platform on Biodiversity and Ecosystem Services, IPBES (Díaz et al. 2015) are acknowledging that institutional and governance structures are the central ingredient of the link between ecosystems and human well-being. Recent work calling for more critical assessment of the role of social power relations and institutions within the ecosystem services framework (Berbés-Blázquez et al. 2016) opens a door for greater application of intersectional approaches to meet this need, especially for addressing gendered power dynamics in the co-production and distribution of ecosystem services. Applying an intersectional lens to the ecosystem services approach would further enhance the role of institutional analysis of the supply and demand of ecosystem services by considering the role power dynamics in shaping trade-offs among ecosystem user-groups (Berbés-Blázquez et al. 2016). This calls for ecosystem service assessments to expand their current methodological toolkit by means of, for example, LIG or participatory scenario planning approaches as a way of tracking social–ecological resilience to climate change.

Integration of explicit mapping of ecosystem service flows associated with differentiated gender roles within a landscape, under an intersectionality prism, would also contribute to emerging lines of work on, for example, gender-sensitive ideas of climate-smart agriculture (CSA). CSA serves as an umbrella approach for those efforts aimed at tackling combined challenges of climate change and food security. In fact, within CSA, gender is already being mainstreamed but equity and power relations have yet to be sufficiently dealt with (Bernier et al. 2013). Some recent work on CSA and gender is, however, beginning to recognize broader social dimensions affecting vulnerability than those easily associated with men or women (Beuchelt and Badstue 2013; Aryal et al. 2014). In FAO’s 2016 publication on “Gender integration into climate-smart agriculture,” the authors highlight the heterogeneity among and within gender groups and describe a selection of quantitative and qualitative techniques that may help in gathering differentiated data. This progress serves to highlight the increasing awareness of these issues and should be supported moving forward. Integrating intersectional perspectives with participatory and feminist GIS and a socially and institutionally charged ecosystem services approach may be useful for building on current gender-focused CSA work and at the same time for developing more holistic understandings of vulnerability and adaptive capacity of farmers across agrarian landscapes. This is seen as especially relevant since the landscape level of adaptation programming is seen as a key component of CSA.

## Conclusion

There is a need for matching the gender framings within research and programming focused on vulnerability, resilience, and adaptation with those being put forth within contemporary feminist studies. Accomplishing this means moving beyond solely using gender categories of ‘men’ and ‘women’ as privileged social divisions that serve to maintain the received wisdom and simplistic priori assumptions about gender categories and vulnerability to climate change which seldom is backed with careful empirical investigation.

We have explored the concept of intersectionality and the potential we see for applying intersectional approaches to gain more nuanced understandings of converging social dimensions that influence exposure, sensitivity, and adaptive capacity to global change, including climate change, especially in the context of agrarian settings in the Global South. We have discussed how we see intersectional understandings contributing to more targeted adaptation actions within local agrarian settings, and how those understandings may be applied to understanding vulnerability and adaptation strategies of agroecosystems at broader landscape levels.

We have argued that that intersectional approaches can be utilized for building more comprehensive understandings of how social dimensions of gender, identity, power, governance, and institutions intersect within different people living in different ecological, economic, and climate contexts to produce webs of distinct exposures, sensitivities, and adaptive capacities. Since these webs are dynamic, it is anticipated that their uptake within a broader variety of climate-related disciplines and greater integration with cutting edge technologies will help to better inform future adaptation actions across agrarian landscapes in the Global South. The necessity of developing new, transformative, and flexible strategies for collaborative and co-designed adaptation interventions that merge the expertise, experience, and influence of a range of different actors (e.g., farmers, extension workers, policy makers, private sector actors) cannot be emphasized enough. This flexibility, it is hoped, will help to minimize trade-offs and promote synergies between place-specific localized adaptation programs, and broader efforts aimed at system-level resilience to climate change and, in turn, this will help steer strategic thinking on sustaining the futures of social–ecological resilience in agroecosystems.

## Footnotes

1 Here we refer to methods such as semi-structured interviews and focus group discussions employed through the Livelihoods as Intimate Government (LIG) approach that is discussed in the last section of this paper.

## References

[CR1] Adger WN (1999). Social vulnerability to climate change and extremes in coastal Vietnam. World Development.

[CR2] Adger WN (2006). Vulnerability. Global Environmental Change.

[CR3] Adger, W. N. 2010. Social Capital, Collective Action, and Adaptation to Climate Change. In *Der Klimawandel*, 327–45. Springer, Fachmedien Wiesbaden GmbH, Wiesbaden. http://link.springer.com/chapter/10.1007/978-3-531-92258-4_19.

[CR4] Adger WN, Brown K, Nelson DR, Berkes F, Eakin H, Folke C, Galvin K, Gunderson L (2011). Resilience implications of policy responses to climate change. Wiley Interdisciplinary Reviews: Climate Change.

[CR5] Agrawal, A. 2010. Local Institutions and Adaptation to Climate Change. In: Mearns and Norton. 2010. *Social Dimensions of Climate Change: Equity and Vulnerability in a Warming World,* World Bank Document. 173–198.

[CR6] Agarwal B (1992). The gender and environment debate: Lessons from India. Feminist Studies.

[CR7] Aitken SC, Michel SM (1995). Who contrives the ‘real’ in GIS? Geographic information, planning and critical theory. Cartography and Geographic Information Systems.

[CR8] Arndt C, Tarp F (2000). Agricultural technology, risk, and gender: A CGE analysis of Mozambique. World Development.

[CR9] Aryal S, Cockfield G, Maraseni TN (2014). Vulnerability of Himalayan transhumant communities to climate change. Climatic Change.

[CR10] Babugura A, Mtshali NC, Mtshali M (2010). Gender and climate change: South Africa case study.

[CR11] Banerjee, A. V., and E. Duflo. 2009. The experimental approach to development economics. Annual Review of Economics, 1:151-178. Downloaded from www.annualreviews.org Access provided by Massachusetts Institute of Technology (MIT) on 10/22/15http://www.nber.org/papers/w14467.

[CR12] Barry H, Schlegel A (1982). Cross-cultural codes on contributions by women to subsistence. Ethnology.

[CR14] Berbés-Blázquez M, González JA, Pascual U (2016). Towards an ecosystem services approach that addresses social power relations. Current Opinion in Environmental Sustainability.

[CR15] Bernard E, Barbosa L, Carvalho R (2011). Participatory GIS in a sustainable use reserve in Brazilian Amazonia: Implications for management and conservation. Applied Geography.

[CR16] Bernier, Q., P. Franks, P. Kristjanson, H. Neufeldt, A. Otzelberger, and K. Foster. 2013. Addressing gender in climate-smart smallholder agriculture. A brief from the CGIAR program on Climate Change, Agriculture and Food Security https://cgspace.cgiar.org/handle/10568/27836.

[CR17] Beuchelt TD, Badstue L (2013). Gender, nutrition-and climate-smart food production: Opportunities and trade-offs. Food Security.

[CR18] Brody A, Demetriades J, Esplen E (2008). Gender and climate change: Mapping the linkages, a scoping study on knowledge and gaps.

[CR19] Buechler S, Hanson A-M, Liverman D, Gay-Antaki M (2015). Advancing multi-disciplinary scholarship on gender, water, and environmental change through feminist political ecology. A Political Ecology of Women, Water and Global Environmental Change..

[CR20] Caldecott L, Leland S (1983). Reclaim the earth women speak out for life on Earth.

[CR21] Carr ER (2008). Men’s crops and women’s crops: The importance of gender to the understanding of agricultural and development outcomes in Ghana’s central region. World Development.

[CR22] Carr ER (2013). Livelihoods as intimate government: Reframing the logic of livelihoods for development. Third World Quarterly.

[CR23] Carr ER (2014). From description to explanation: Using the livelihoods as intimate government (LIG) approach. Applied Geography.

[CR24] Carr, E. R., and M. C. Thompson. 2013. Gender and climate change adaptation in agrarian settings. Report prepared for the United States Agency for International Development. pp. 76.

[CR25] Carr ER, Thompson MC (2014). Gender and climate change adaptation in agrarian settings: Current thinking, new Directions, and research Frontiers. Geography Compass.

[CR26] Carr ER, Owusu-Daaku KN (2016). The shifting epistemologies of vulnerability in climate services for development: The case of Mali’s agrometeorological advisory programme. Area.

[CR27] Carr ER, Fleming G, Tshibangu K (2016). Understanding women’s needs for weather and climate information in agrarian settings: The case of Ngetou Maleck, Senegal. Weather, Climate, and Society..

[CR28] Carr, E. R., S. Onzere, T. Kalala, K.N. Owusu-Daaku, and H. Rosko. 2015a. Assessing Mali’s l’Agence Nationale de la Météorologie’s (Mali Meteo) Agrometeorological Advisory Program: Final report in the farmer use of advisories and the implications for climate service design. Washington, DC.

[CR29] Carr ER, Abrahams D, De la Poterie AT, Suarez P, Koelle B (2015). Vulnerability assessments, identity and spatial scale challenges in disaster-risk reduction. Jàmbá: Journal of Disaster Risk Studies.

[CR30] Chant S, Chant S (2010). Gendered poverty across space and time: introduction and overview. The international handbook of gender and poverty: Concepts, research, policy.

[CR31] Cornwall A, Harrison E, Whitehead A (2007). Gender myths and feminist fables: The struggle for interpretive power in gender and development. Development and Change.

[CR32] Cornwall A, Rivas AM (2015). From ‘gender equality and ‘women’s empowerment’ to global justice: reclaiming a transformative agenda for gender and development. Third World Quarterly.

[CR33] Crenshaw K (1991). Mapping the margins: Intersectionality, identity politics, and violence against women of Color. Stanford Law Review.

[CR34] Dankelman I, Jansen W, Dankelman I (2010). Gender, environment, and climate change: Understanding the linkages. Gender and climate change: An introduction.

[CR35] Davis K (2008). Intersectionality as buzzword: A sociology of science perspective on what makes a feminist theory successful. Feminist Theory.

[CR36] Demetriades J, Esplen E (2010). The gender dimensions of poverty and climate change adaptation. IDS Bulletin.

[CR37] Díaz S, Demissew S, Carabias J, Joly C, Lonsdale M, Ash N, Larigauderie A, Adhikari Jay Ram (2015). The IPBES conceptual framework: Connecting nature and people. Current Opinion in Environmental Sustainability.

[CR38] Djoudi H, Brockhaus M (2011). Is adaptation to climate change gender neutral? Lessons from communities dependent on livestock and forests in northern Mali. International Forestry Review.

[CR39] Elwood S (2008). Volunteered geographic information: Future research directions motivated by critical, participatory, and feminist GIS. GeoJournal.

[CR40] Evans R (2016). Gendered struggles over land: Shifting inheritance practices among the Serer in rural Senegal. Gender, Place & Culture.

[CR41] FAO. 2011. The state of food and agriculture 2010–2011: Women in agriculture closing the gender gap for development. Rome. [Online]. Retrieved from: http://www.fao.org/docrep/013/i2050e/i2050e00.htm.

[CR42] FAO. 2016. Gender Integration into Climate Smart Agriculture. Tools for Data Collection and Analysis for policy and research. Retrieved from: http://www.fao.org/3/a-i5299e.pdf.

[CR43] Fisher, S. 2014. Tracking adaptation and measuring development through a gender lens. IIED Briefing Paper. http://pubs.iied.org/pdfs/17270IIED.pdf.

[CR44] Fordham MH (1998). Making women visible in disasters: Problematising the private domain. Disasters.

[CR45] Harding, S., 1995. Just add women and stir? In *UN Gender Working Group Book on the Overlay of Science and Technology [S&T], sustainable human development, and gender issues*, In: Missing links: gender equity in science and technology for development, [compiled by] United Nations. Commission on Science and Technology for Development. Gender Working Group. Ottawa, Canada, International Development Research Centre [IDRC], 1995. 295–307. Retrieved from: http://popline.org/node/308122.

[CR46] Harman, M. 2013. Using qualitative Geographic Information Systems to explore gendered dimensions for conservation agriculture production systems in the Philippines: A Mixed Methods Approach. Virginia Tech. https://vtechworks.lib.vt.edu/handle/10919/50811.

[CR47] Harvey F, Kwan M-P, Pavlovskaya M (1998). Introduction: critical GIS. Cartographica: The International Journal for Geographic Information and Geovisualization.

[CR48] Hooks B (1984). Ain’t I a woman: Black women and feminism.

[CR49] IPCC, Intergovernmental Panel on Climate Change. 2014. IPCC WGII AR5 Climate Change 2014: Impacts, Adaptation, and Vulnerability. Technical Summary.

[CR50] Jackson C (1998). Gender, irrigation, and environment: arguing for agency. Agriculture and Human Values.

[CR51] Kaijser A, Kronsell A (2014). Climate change through the lens of intersectionality. Environmental Politics.

[CR52] Kambic K (2015). Urban water visibility in Los Angeles: Legibility and access for all. Chapter four In: Buechler, Stephanie, Anne-Marie Hanson, Diana Liverman, and Miriam Gay-Antaki. 2015. Advancing multi-disciplinary scholarship on gender, water, and environmental change through feminist political ecology. A Political Ecology of Women, Water and Global Environmental Change.

[CR53] Karlan D, Zinman J (2011). Microcredit in theory and practice: Using randomized credit scoring for impact evaluation. Science.

[CR54] Kwan M-P (2002). Feminist visualization: Re-envisioning GIS as a method in feminist geographic research. Annals of the Association of American Geographers.

[CR55] Kwan M-P (2002). Is GIS for women? Reflections on the critical discourse in the 1990s. Gender, Place and Culture: A Journal of Feminist Geography.

[CR56] Leach M (2007). Earth mother myths and other ecofeminist fables: How a strategic notion rose and fell. Development and change.

[CR57] Liverman, D.M. 1990. Vulnerability to global environmental change. In *Understanding Global Environmental Change: The Contributions of Risk Analysis and Management: A Report on an International Workshop, October 11–13, 1989*, ed. R.E. Kasperson, 27–44. Worcester: Clark University.

[CR58] Lykke, N. 2011. Intersectional analysis: Black box or useful critical feminist thinking technology. In *Framing Intersectionality: Debates on a Multi*-*Faceted Concept in Gender Studies*, ed. H. Lutz, M.T. Herrera Vivar, and L. Supik, 207–220. Burlington: Ashgate Publishing Company.

[CR59] McCall MK (2003). Seeking good governance in participatory-GIS: A review of processes and governance dimensions in applying GIS to participatory spatial planning. Habitat International.

[CR60] McLafferty S (2005). Women and GIS: Geospatial technologies and feminist geographies. Cartographica: The International Journal for Geographic Information and Geovisualization.

[CR100] Mehra, R., and M. Hill Rojas. 2008. A significant shift: Women, food security and agriculture in a global marketplace. International Center for Research on Women (ICRW) citing FAO focus on women and food security. Rome: Food and Agriculture Organization of the United Nations. http://www.icrw.org/publications/women-food-security-and-agriculture-global-marketplace.

[CR61] Mies M, Shiva V (1993). Ecofeminism.

[CR62] Nelson DR, Adger WN, Brown K (2007). Adaptation to environmental change: Contributions of a resilience framework. Annual Review of Environment and Resources.

[CR63] Nightingale AJ (2011). Bounding difference: Intersectionality and the material production of gender, caste. Class and Environment in Nepal. Geoforum.

[CR64] Patt, A.G., A. Dazé, and P. Suarez. 2009. Gender and climate change vulnerability: What’s the problem, what’s the solution. In *The Distributional Effects of Climate Change: Social and Economic Implications*, ed. M. Ruth, and M.E. Ibarrarán, 82–102. Cheltenham: Edward Elgar Publishing.

[CR65] Paavola J, Adger WN (2006). Fair adaptation to climate change. Ecological Economics.

[CR66] Peet R, Watts M (2004). Liberation ecologies: Environment, development, social movements.

[CR67] Pelling M, High C (2005). Understanding adaptation: What can social capital offer assessments of adaptive capacity?. Global Environmental Change.

[CR68] Quisumbing, A.R., and L. Pandolfelli. 2008. *Promising approaches to address the needs of poor female farmers*. International Food Policy Research Institute. IFPRI Discussion Paper 00882: 1–29.

[CR69] Ravera F, Martín-López B, Pascual U, Drucker A (2016). The diversity of gendered adaptation strategies to climate change of Indian farmers: A feminist intersectional approach. Ambio.

[CR70] Reid P, Vogel C (2006). Living and responding to multiple stressors in South Africa—Glimpses from KwaZulu-Natal. Global Environmental Change.

[CR71] Ribot J (2011). Vulnerability before adaptation: Toward transformative climate action. Global Environmental Change.

[CR72] Rodó-de-Zárate M (2014). Developing geographies of intersectionality with relief maps: Reflections from youth research in Manresa, Catalonia. Gender, Place & Culture.

[CR73] Seager J (1993). Earth follies: Feminism, politics and the environment.

[CR74] Sui D, DeLyser D (2012). Crossing the qualitative-quantitative chasm I hybrid geographies, the spatial turn, and volunteered geographic information (VGI). Progress in Human Geography.

[CR75] Sultana F (2010). Living in hazardous waterscapes: Gendered vulnerabilities and experiences of floods and disasters. Environmental Hazards.

[CR76] Sultana F (2014). Gendering climate change: Geographical insights. The Professional Geographer.

[CR77] Tatlonghari GT, Paris TR (2013). Gendered adaptations to climate change: A case study from the Philippines. Research, Action and Policy: Addressing the Gendered Impacts of Climate Change.

[CR78] Turner BL, Kasperson RE, Matson PA, McCarthy JJ, Corell RW, Christensen L, Eckley N, Kasperson JX (2003). A framework for vulnerability analysis in sustainability science. Proceedings of the National Academy of Sciences.

[CR79] Tyrväinen L, Mäkinen K, Schipperijn J (2007). Tools for mapping social values of urban woodlands and other green areas. Landscape and Urban Planning.

[CR80] Walker B, Gunderson L, Kinzig A, Folke C, Carpenter S, Schultz L (2006). A Handful of heuristics and some propositions for understanding resilience in social-ecological systems. Ecology and Society.

[CR81] Winowiecki, L., C. Mwongera, and F. Rubiano. 2014. Climate smart agriculture rapid appraisal (CSA-RA): A prioritization tool for outscaling. https://cgspace.cgiar.org/handle/10568/65658.

[CR83] UN DESA, 2015. United Nations Department of economic and social affairs transforming our world: The 2030 agenda for sustainable development. https://sustainabledevelopment.un.org/post2015/transformingourworld.

[CR84] United Nations. 2016. Sustainable development knowledge platform. https://sustainabledevelopment.un.org.

[CR85] Valentine G (2007). Theorizing and researching intersectionality: A challenge for feminist geography. The Professional Geographer..

[CR86] Villamor GB, Palomo I, Santiago CAL, Oteros-Rozas E, Hill J (2014). Assessing stakeholders’ perceptions and values towards social-ecological systems using participatory methods. Ecological Processes.

